# Supplementary figures and images for: Oligosaccharyltransferase (OST) complex inhibition effectively treats rodent and human prions
Source: PLoS Pathog. 2026 Jan 12;22(1):e1013867. doi: 10.1371/journal.ppat.1013867 (PMC12818742; doi:10.1371/journal.ppat.1013867)

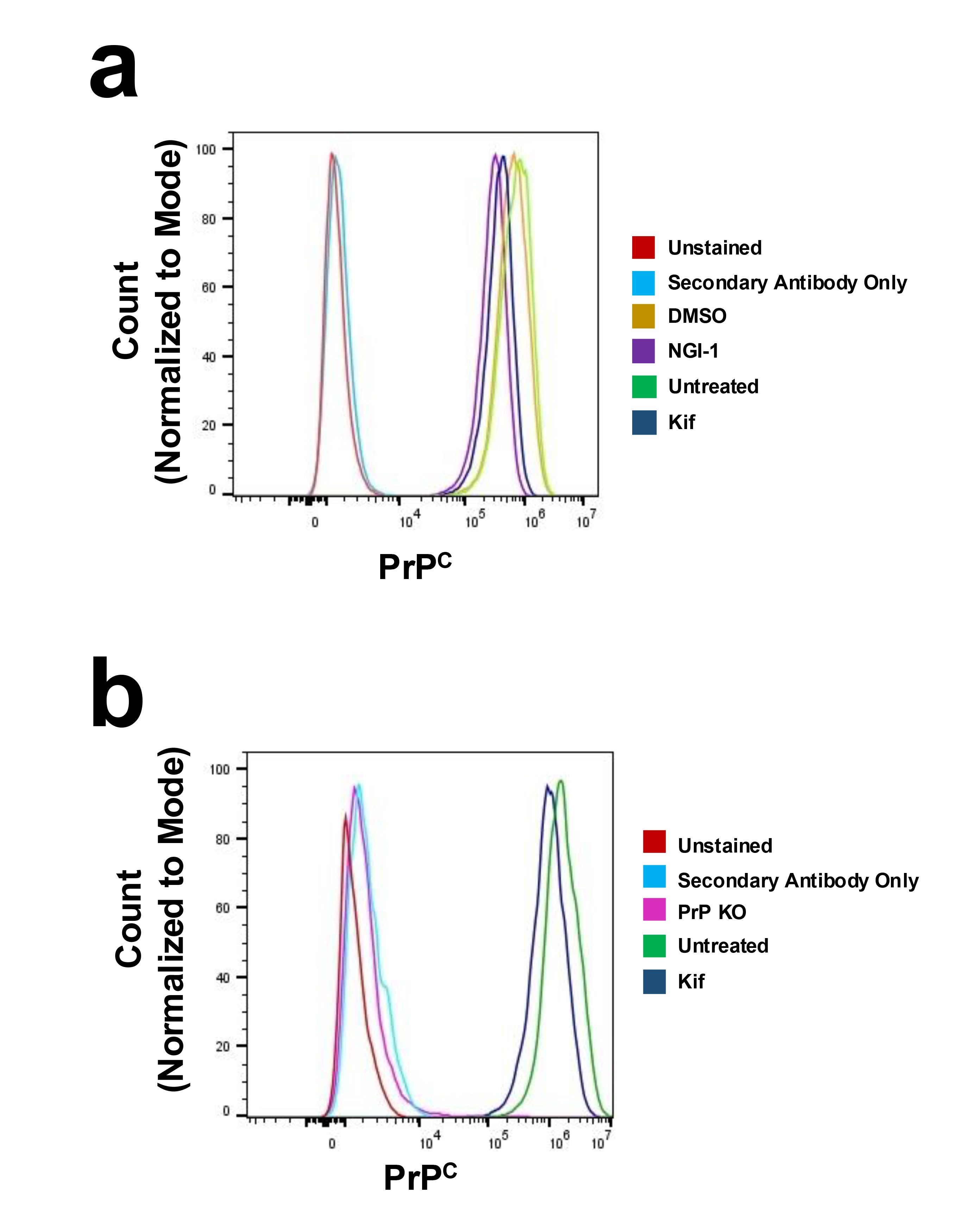

Supplement: S1 Fig — (A) Representative flow cytometry plot for PrPC surface expression in undifferentiated CAD5 cells treated with 5 μM NGI-1, 5 μM kifunensine, or vehicle equivalent for 72 hr. (B) Representative flow cytometry plot for PrPC surface expression in undifferentiated CAD5 cells treated with 10 μM kifunensine or vehicle equivalent for 48 hr. (TIF) [file ppat.1013867.s001.tif]

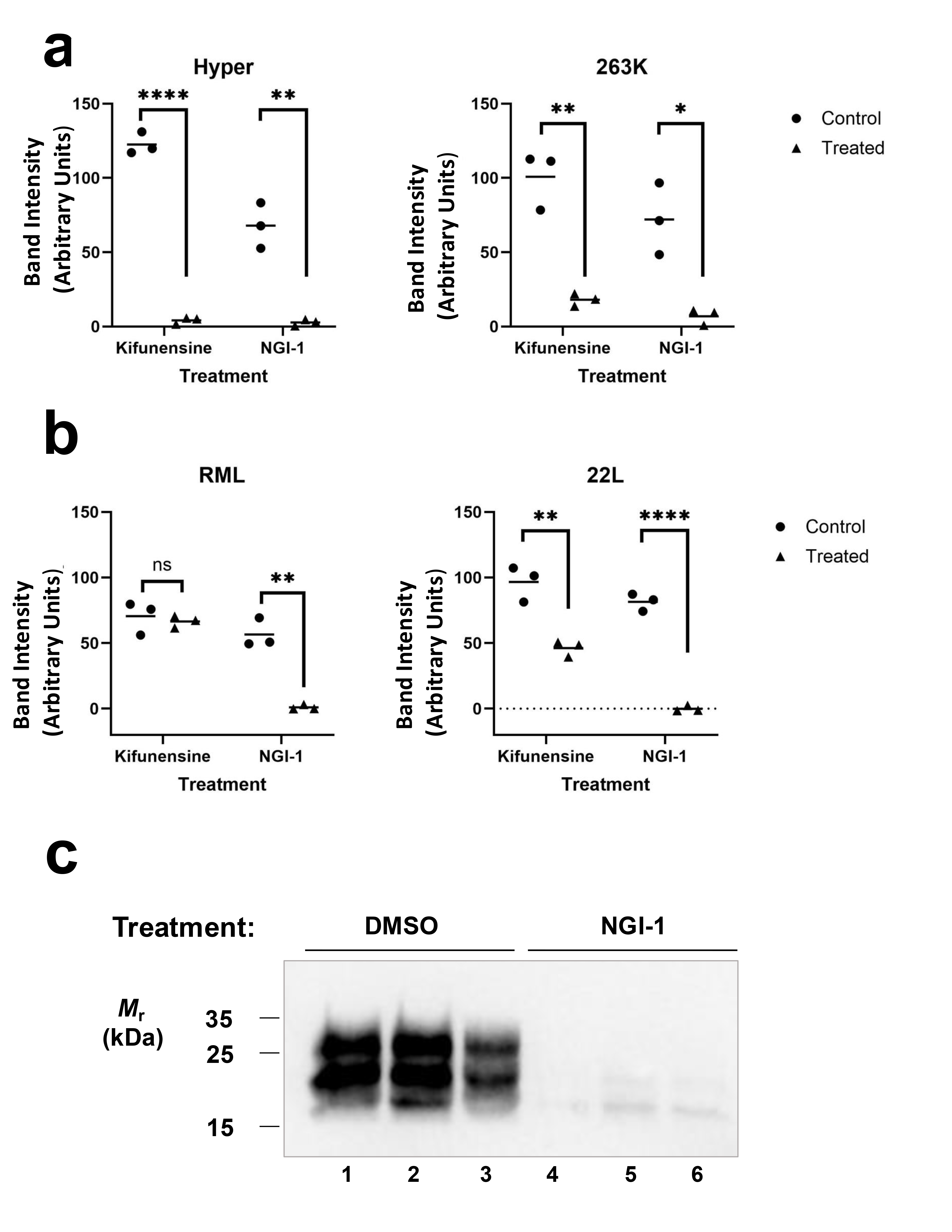

Supplement: S2 Fig — (A) Quantification of Western blots shown in (Fig 1C). (B) Quantification of Western blot shown in (Fig 1D). Asterisks represent significance values from unpaired t-tests as follows: *p ≤ 0.05, **p ≤ 0.01, ****p ≤ 0.0001, ns = not significant. (C) Western blot for PK-sensitive conformations of PrPSc. Phosphotungstic acid (PTA) precipitation was performed to isolate PrPSc from lysates of CAD5-RML cells treated for 5 days with 5 μM NGI-1 or DMSO. [Subxref0](TIF)[Subxref0] [file ppat.1013867.s002.tif]

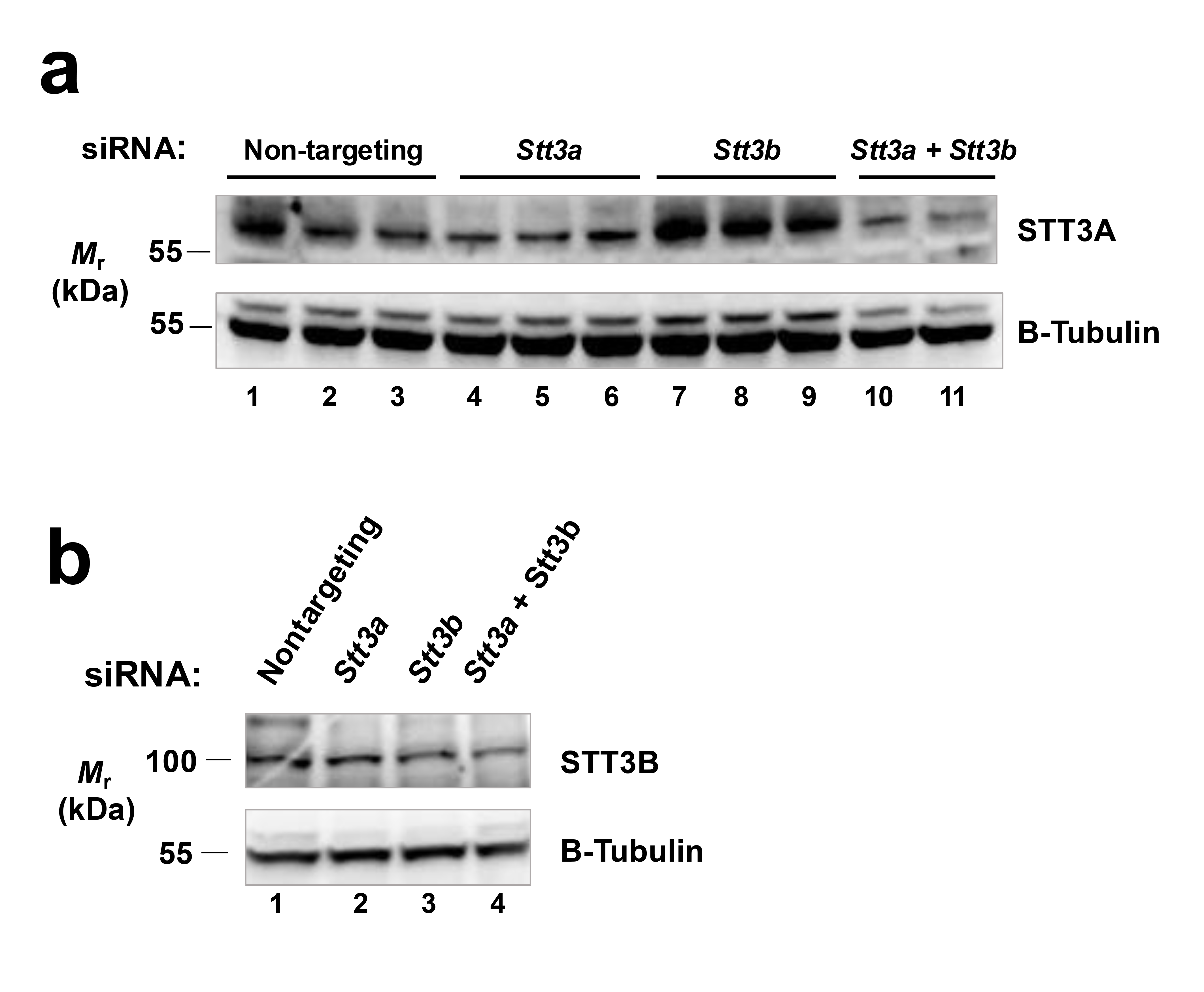

Supplement: S3 Fig — (A) Western blot showing effect of siRNA knockdown of Stt3a and/or Stt3b on STT3A protein levels in CAD5-22L cell lysate. (B) Western blot showing effect of siRNA knockdown of Stt3a and/or Stt3b on STT3B protein levels in CAD5-22L cell lysate. (TIF) [file ppat.1013867.s003.tif]

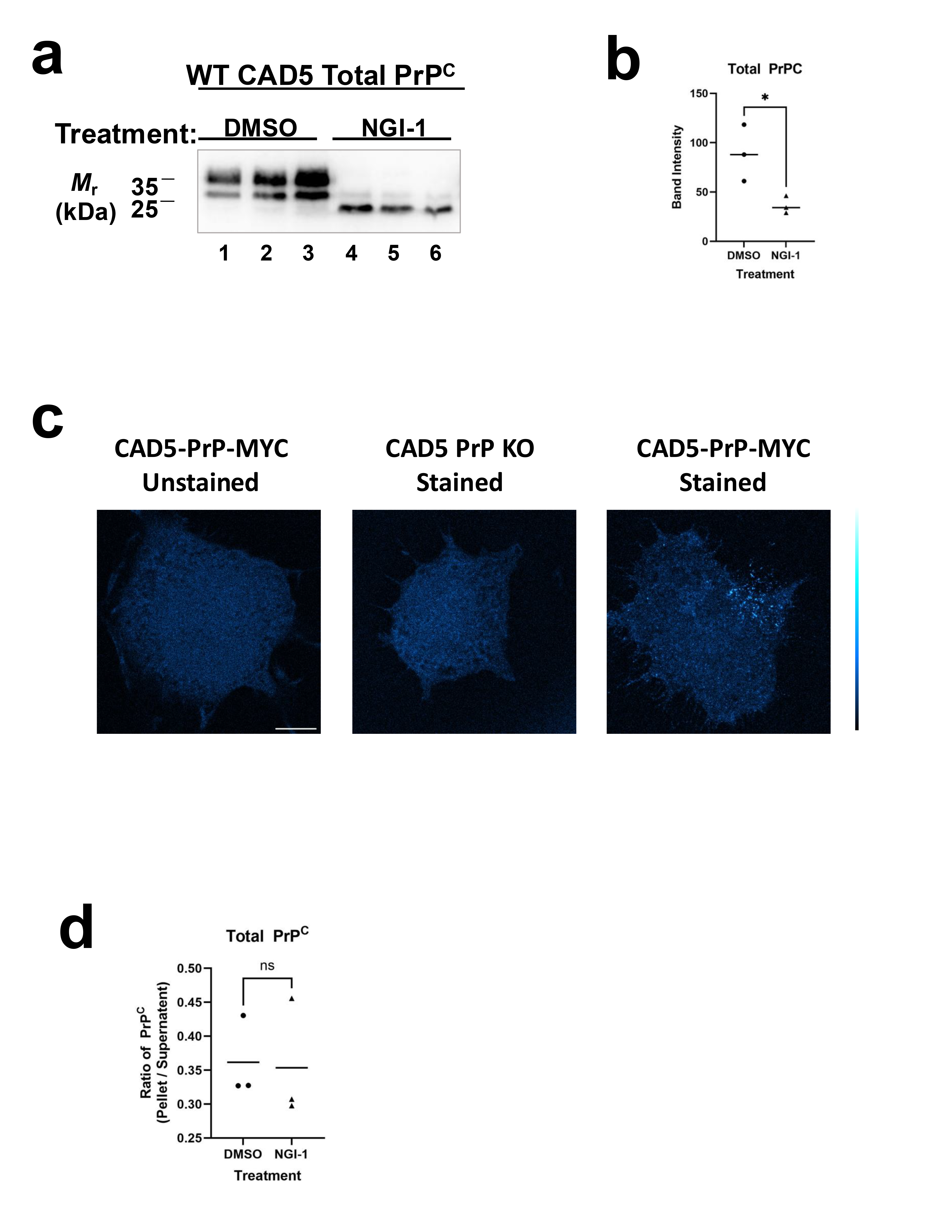

Supplement: S4 Fig — (A) Western blot showing total PrP in lysates from undifferentiated CAD5 cells treated for 1 week with 5 μM NGI-1 or DMSO. (B) Quantification of the Western Blot shown in (A). (C) Single channel (488) confocal images showing autofluorescence of unstained CAD5-PrP-MYC cells (left panel) or stained CAD5-PrP-KO cells (middle panel) and presence of PrP puncta in stained CAD5-PrP-MYC cells (right panel). All cells were fixed with 4% paraformaldehyde and stained samples (middle and right panels) were stained with anti-MYC antibody to visualize PrP. Scale bar, 10 μm. (D) Quantification of the Western blot in Fig 3C comparing the proportion of total PrP found in the pellet and supernatant across treatments. Points represent individual samples from biological triplicates. Asterisks represent significance values from unpaired t-tests as follows: *p ≤ 0.05, ns = not significant. (TIF) [file ppat.1013867.s004.tif]

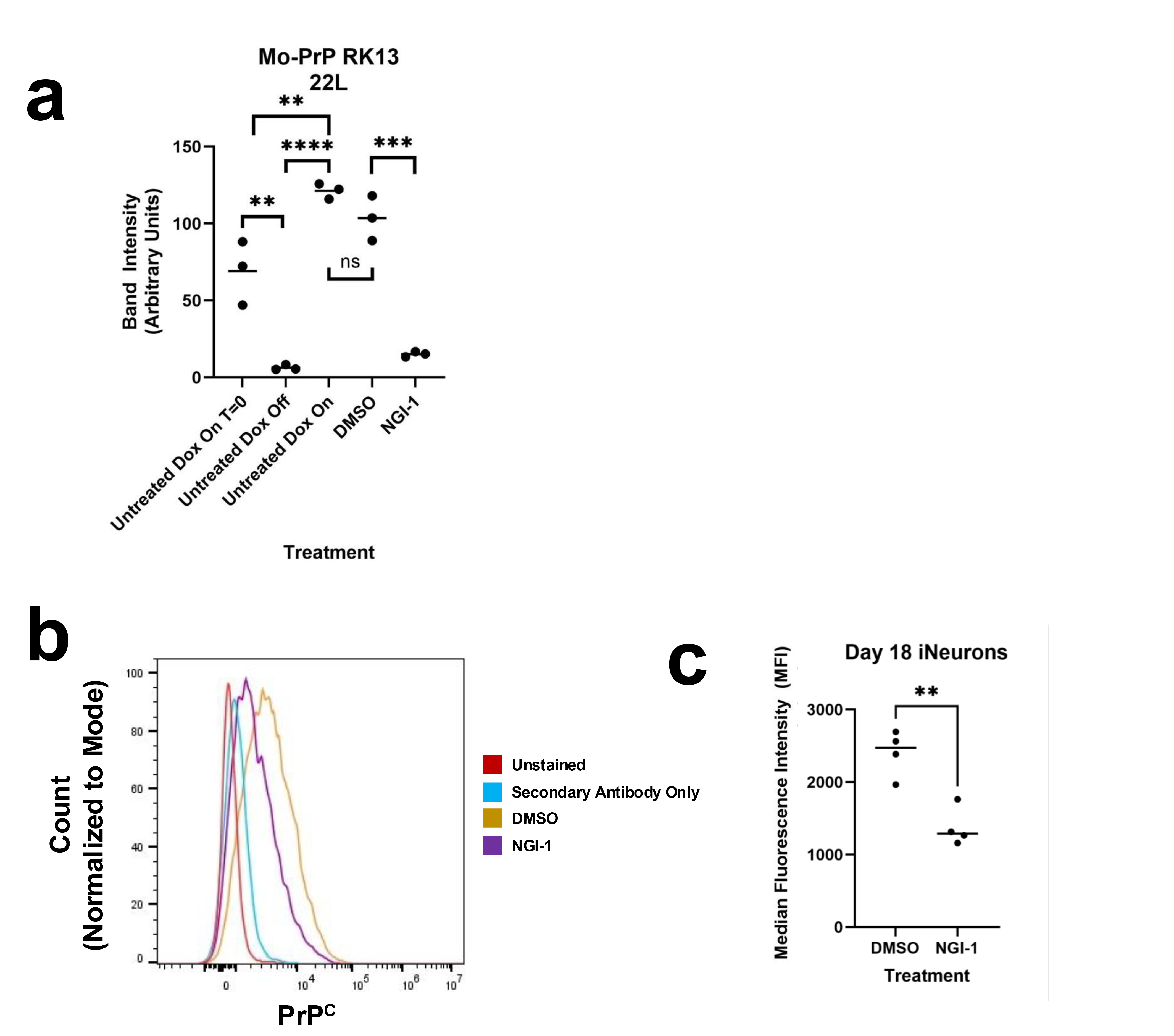

Supplement: S5 Fig — (A) Quantification of Western blot shown in Fig 4A. (B) Representative flow cytometry plot showing reduction of surface PrPC in NGI-1-treated i3Neurons (C) Quantification of flow cytometry data in (B). Points represent individual samples from biological quadruplicate. Day 15 i3Neurons were treated with 5 μM NGI-1 or vehicle equivalent for 72 hr. Asterisks represent significance values from unpaired t-tests as follows: **p ≤ 0.01, ***p ≤ 0.001, ****p ≤ 0.0001, ns = not significant. (TIF) [file ppat.1013867.s005.tif]

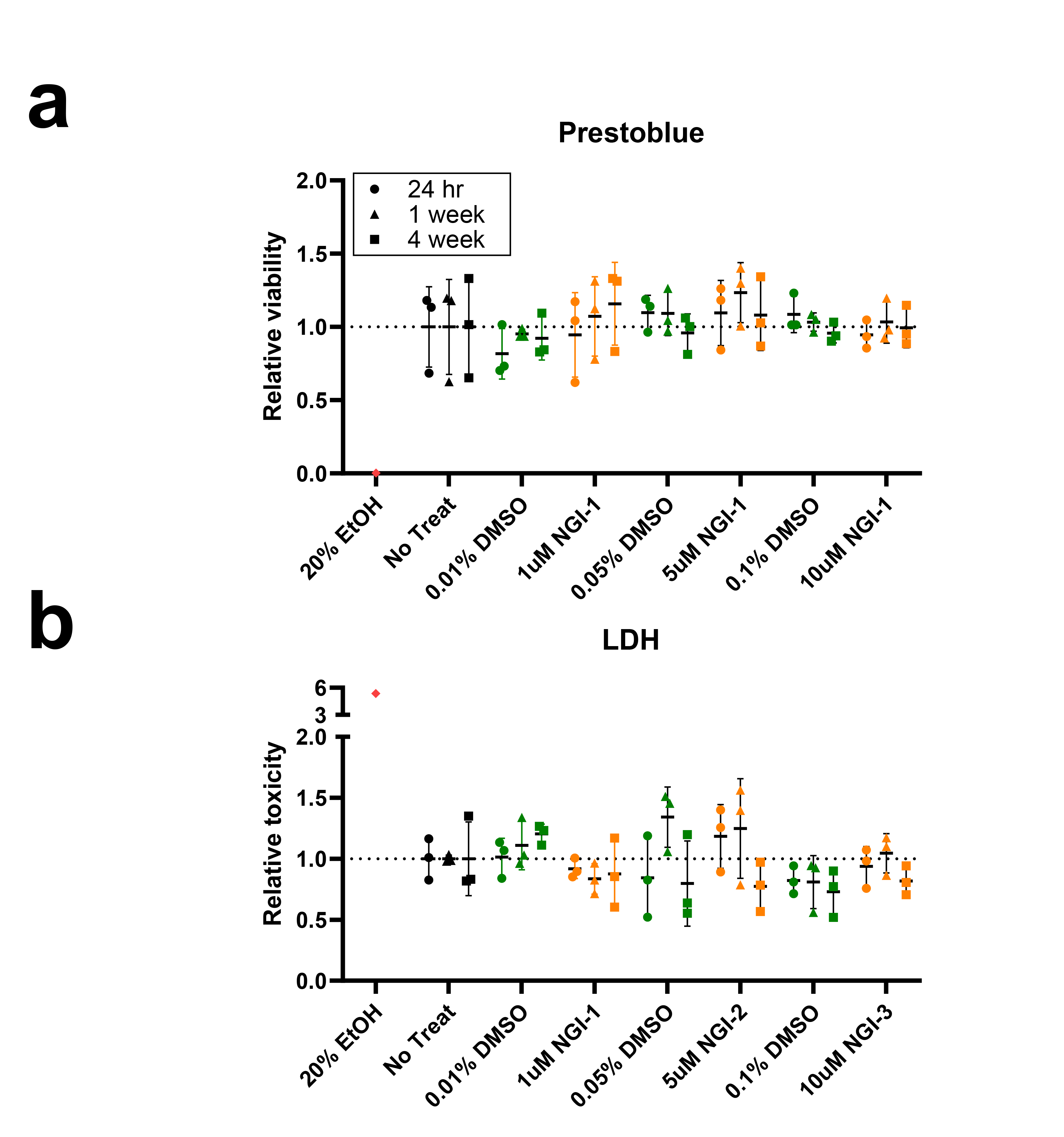

Supplement: S6 Fig — (A) PrestoBlue and (B) LDH assays were performed on organoids (n = 3 per condition) at 24 hr (circles), 1 week (triangles), and 4 weeks (squares) following continued treatment with indicated concentrations of DMSO (dark yellow), NGI-1 (purple), or no treatment (black). A 5 min 20% EtOH control treatment (red diamond) is also shown. Data are normalized to the average of the no treatment controls (dotted line). Each marker represents an individual organoid. (TIF) [file ppat.1013867.s006.tif]

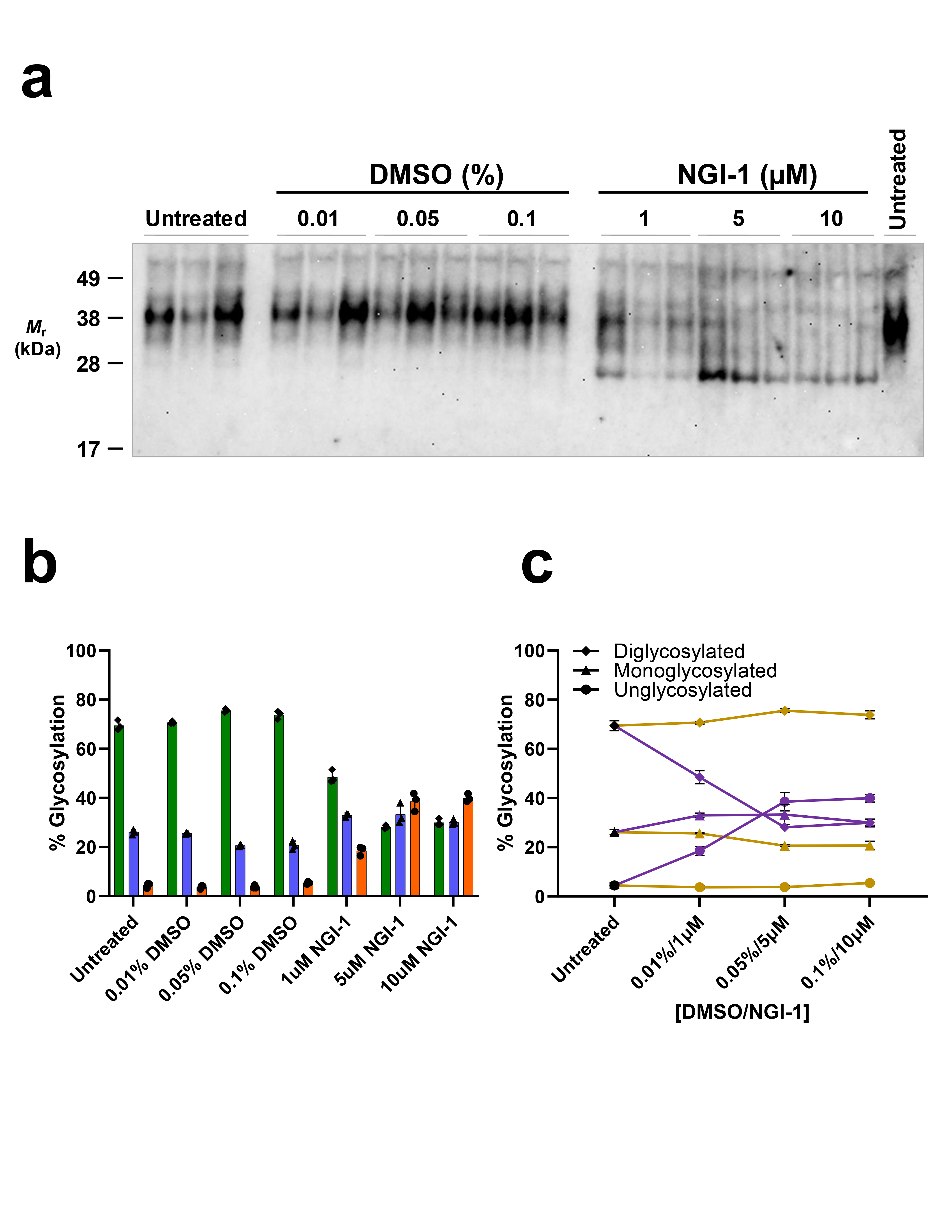

Supplement: S7 Fig — (A) Western blot probed with anti-PrP 3F4 antibody of organoids treated for 30 days with the indicated concentrations of DMSO or NGI-1. (B&C) Quantitation of glycosylation bands from A showing the percent of di- (diamonds), mono- (triangles), or unglycosylated (circles) PrP for each sample. Each marker represents a single organoid (n = 3 per condition). (TIF) [file ppat.1013867.s007.tif]

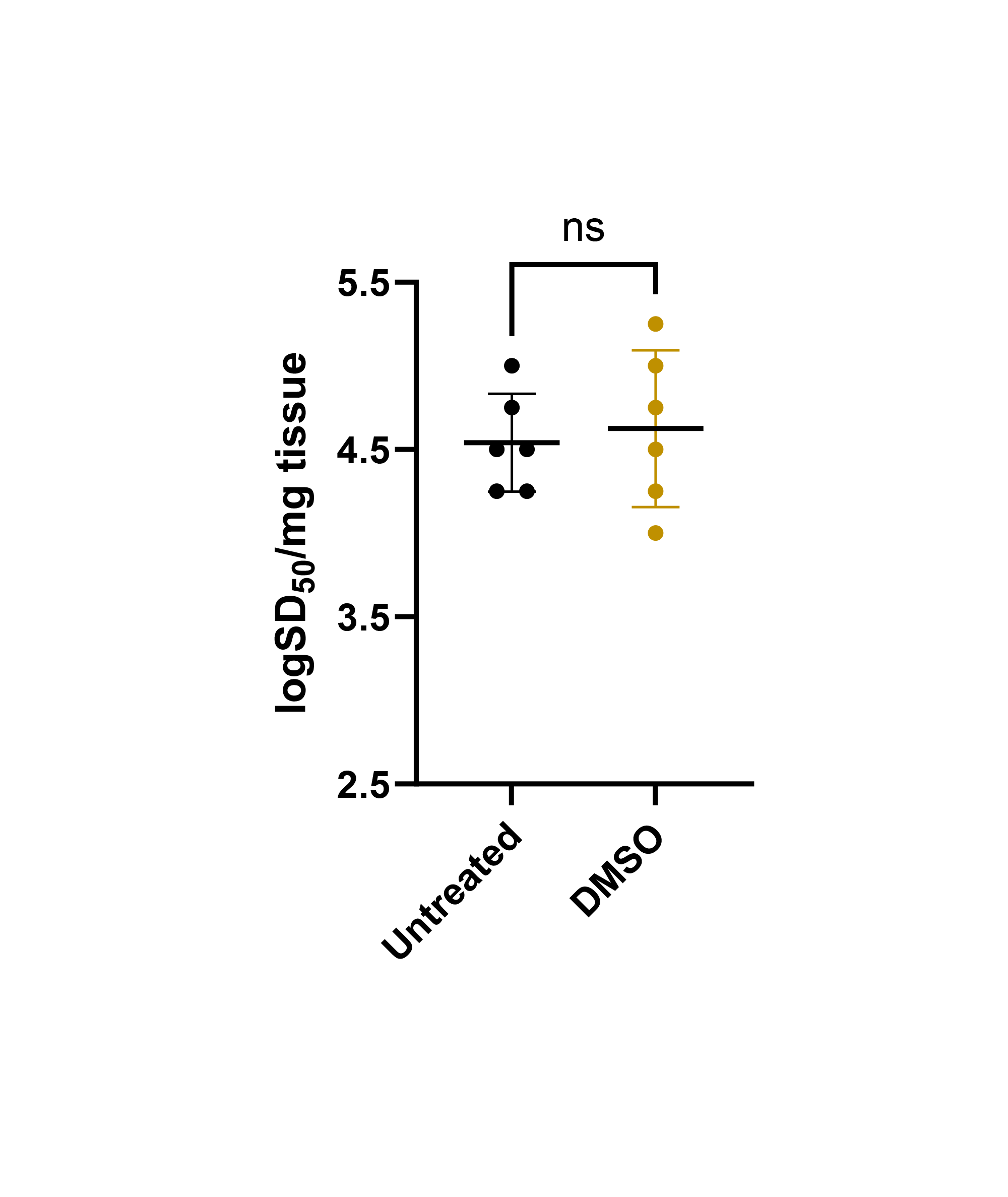

Supplement: S8 Fig — (A) CJD-infected organoids with or without a 30-day treatment with 0.1% DMSO treatment (0.1%) were collected at ~120dpi and subjected to RT-QuIC end-point dilution analysis and seeding activity quantitation by Spearman-Kärber analyses. Each marker represents data from an individual organoid (n = 6) and ns means not significant by Mann Whitney test. (TIF) [file ppat.1013867.s008.tif]
